# Exploring Orodispersible Films Containing the Proteolysis Targeting Chimera ARV-110 in Hot Melt Extrusion and Solvent Casting Using Polyvinyl Alcohol

**DOI:** 10.3390/pharmaceutics16121499

**Published:** 2024-11-22

**Authors:** Valentina Meloni, Laura Halstenberg, Lena Mareczek, Jankin Lu, Bonnie Liang, Nadine Gottschalk, Lena K. Mueller

**Affiliations:** 1 Merck Life Science KGaA, 64293 Darmstadt, Germany; valentina.meloni@merckgroup.com (V.M.); lena.mueller@merckgroup.com (L.K.M.); 2 Merck Chemicals (Shanghai) Co., Ltd., Shanghai 201203, China

**Keywords:** PVA, Orodispersible films, ODF, PROTACs, amorphous solid dispersions, ASD, MeltPrep

## Abstract

Background/Objectives: This project aims to provide valuable insights into the formulation of orodispersible films (ODFs) for the delivery of PROTAC ARV-110. The primary objective of this drug delivery formulation is to enhance the solubility of PROTAC ARV-110, which faces significant challenges due to the low solubility of this active pharmaceutical ingredient, as it belongs to a molecular class that is considered to exceed the “Rule of Five”. Methods: We employed the concept of developing a rapidly disintegrating ODF to enhance the solubility of PROTAC ARV-110, utilizing polyvinyl alcohol as the polymer of choice. Given the high thermal stability of ARV-110, the PROTAC was subjected to two primary ODF manufacturing techniques: Hot melt extrusion (HME) and solvent casting. To establish the HME method, pre-screening through vacuum compression molding was performed. The films were characterized based on their disintegration in artificial saliva, drug release in a physiological environment, and mechanical strength. Results: All formulations demonstrated enhanced solubility of ARV-110, achieving exceptional results in terms of disintegration times and resistance to applied stress. Conclusions: The findings from the experiments outlined herein establish a solid foundation for the successful production of orodispersible films for the delivery of PROTACs.

## 1. Introduction

The oral route for pharmaceutical administration has always been and continues to be the preferred route for patients. However, infants and dysphagic patients have difficulties swallowing tablets. Therefore, the use of drug delivery systems such as orodispersible films (ODF) represents an advantageous route of administration for these patients. A further advantage of ODF preparations is that they do not require any water as they quickly dissolve in the mouth when in contact with saliva. Recently, these dosage forms are considered excellent candidates for personalized medicine [1,2,3]. Gupta et al. describe three different types of ODFs, which are classified depending on their dissolving times, layer composition, and source of active pharmaceutical ingredient (API). Based on these characteristics, the films can be used to target different purposes [4].

The excipients used in the formulation of ODFs consist of polymers, generally soluble in water, plasticizers to provide flexibility to the film and facilitate manufacturing, and, ultimately, surfactants, which increase the dissolving properties of the film [5,6]. To address the aforementioned dissolving properties, acids (such as ascorbic acid, citric acid, tartaric acid, malic acid, and lactide acid) can be used in the formulation (up to 10%). They stimulate the production of saliva once the film is in the mouth, thereby increasing the rate of disintegration [7].

The emerging ODF is an efficient drug delivery system (DDS) that provides various advantages on patient compliance, cost-effectiveness, and prompt assistance for emergency medication through an oral and, therefore, non-invasive delivery route. The most common way to manufacture ODFs is by using solvent casting and hot melt extrusion (HME) [8]. The solvent casting is hydrous and one of the oldest methods in the preparation of ODFs. It involves dissolving a polymer, API, and other excipients, such as plasticizers and surfactants, followed by evaporation to form a solid film. The solvent is chosen considering the solubility of the active ingredient and the miscibility of the polymer [9]. It is recommended for thermolabile drugs and where lower temperatures are required by the volatile ingredients [7]. HME is a continuous pharmaceutical process that involves mixing and melting of a polymeric material using rotating screws at a temperature that is above the glass transition temperature (Tg) of the respective polymer. When the purpose is to achieve a molecular-level mixing of the active compounds and thermoplastic binders, polymers, or both, the temperature needs to be above their glass transition temperature (Tg) and eventually above their melting temperature (Tm) [10]. Consequently, this method is only applicable to active ingredients, which are highly thermoresistant. With the HME method, films can be created by mixing the drug, film former, plasticizer, surfactants, and other required excipients in appropriate amounts to create a homogeneous blend. Following this, the blend is introduced into a hopper and conveyed to a heated barrel, where uniform films with thicknesses of less than 1 mm can be produced [11]. Vacuum compression molding (VCM) can serve as an ideal technology for initial screening for HME, especially when material is limited [12]. It is a fusion-based method that, starting from powders, forms solid specimens by melting them under a vacuum. It was demonstrated that this technique is capable of predicting the suitability of a polymeric carrier for the respective API [13]. Therefore, this method represents an efficient material-sparing tool during the initial phase of HME development.

With these technologies, solvent casting and HME, solubility, and bioavailability of poorly water-soluble drugs can be enhanced. The crystal lattice of the active is disrupted, through either melting or dissolution and transferred to an amorphous state, which is stabilized by the added polymer. These formulations are commonly known as amorphous solid dispersions (ASD). Dissolution of an ASD in aqueous media results in the generation of a supersaturated solution of the drug, which will eventually lead to nucleation and recrystallization, but the supersaturated state can be stabilized through polymers in the formulation [14,15,16,17,18].

ARV-110 serves as a notable example of an API with extremely low solubility, as it surpasses the “Rule of Five”. This API is associated with Proteolysis Targeting Chimeras (PROTACs), an emerging, cutting-edge molecule class that is being developed to address undruggable targets. They are heterobifunctional compounds that consist of a ligand of the “protein of interest” (POI), which is connected through a linker to a ligand of an E3 ubiquitin ligase [19]. Their mode of action involves the degradation of the target molecule by bringing the POI in proximity to an E3 ligase, which marks the POI for degradation [20]. These molecules have various advantages, such as requiring lower doses compared to conventional small molecules, as they act catalytically, showing impressive inhibition activity against some drug-resistant targets (e.g., the androgen receptor AR). Moreover, they can target undruggable targets (e.g., the signal transducer and activator of transcription 3, STAT-3, and targeting the degradation of entire proteins, they can influence non-enzymatic functions [19]. The main challenge in developing an oral dosage form with PROTACs is the low solubility of this molecule class, leading to challenges in oral bioavailability [21]. However, studies have been published focusing on enhancing the solubility of certain PROTACs through the manufacturing of ASDs and liquid–solid formulations [22,23,24].

The aim of the project is to produce ODFs for the delivery of the PROTAC ARV-110 through HME and solvent casting. For feasibility testing, the VCM method was applied to evaluate HME applicability. The ODFs were characterized by disintegration, dissolution, x-ray powder diffraction, and tensile strength. As a polymer of choice, polyvinyl alcohol (PVA) was used, as we were able to successfully apply this polymer in the solubility enhancement of ARV-110 through spray-drying in earlier experiments [24].

## 2. Materials and Methods

### 2.1. Materials

Bavdegalutamide (ARV-110) (MedChemExpress, Monmouth Junction, New York City NJ, USA); Parteck^®^ MXP 4-88, PVA 4-88, Poly(vinyl alcohol), (Merck KGaA, Darmstadt, Germany); Triacetin (Merck KGaA, Darmstadt, Germany); Parteck^®^ COAT, PVA 5-88, Poly(vinyl alcohol), (Merck KGaA, Darmstadt, Germany); Parteck^®^ SI 150, Sorbitol, (Merck KGaA, Darmstadt, Germany); Sodium Dodecyl Sulfate, Sodium laurilsulfate, (BASF, Ludwigshafen, Germany); TWEEN^®^ 80, Polysorbate 80, (Merck KGaA, Darmstadt, Germany); Patent blau 85 E131 (BASF, Ludwigshafen, Germany); DMSO Dimethyl sulfoxide, (Merck KGaA, Darmstadt, Germany); Ensure^®^ Supelco (Merck KGaA, Darmstadt, Germany); Artificial saliva for pharmaceutical research (Sigma Aldrich, St. Louis, MO, USA); HCl (BASF, Ludwigshafen, Germany); Na_3_PO_4_ buffer solution (Merck KGaA, Darmstadt, Germany); Acetonitrile (Sigma Aldrich, St. Louis, MO, USA); Formic acid (Sigma Aldrich, St. Louis, MO, USA); Phosphate buffer pH 6.8 (protocol by USP)—United States Pharmacopeia; Milli-Q^®^ Water (Merck Millipore, Burlington, MA, USA).

### 2.2. Methods

#### 2.2.1. Vacuum Compression Molding (VCM)

PVA-ARV mix: 292.5 mg of PVA 4-88 was mixed with 7.5 mg of ARV-110 at 2500 rpm for 5 min in a SpeedMixer (Hauschild, Hamm, Germany). After filling the mixture into the VCM tool, a vacuum was applied to compact the powder. The VCM tool (MeltPrep, Graz, Austria) was heated up to 220 °C for 10 min at high pressure. After cooling, the 20 mm disk was milled in the Spex SamplePrep™ Freezer/Mill™ (Fisher Scientific, Waltham, MA, USA) for 5 min (15 CPS, pre-cool and cool time: 2 min).

PVA-Triacetin-ARV mix: 263.25 mg of PVA 4-88 was mixed with 29.25 mg of triacetin at 2500 rpm for 5 min in the SpeedMixer. The mixture was melted at 220 °C for 10 min at high pressure, and the disk was milled in a Spex SamplePrep™ Freezer/Mill™ for 5 min (15 CPS, pre-cool and cool time: 2 min). Next, 7.5 mg of ARV-110 was added to the PVA-triacetin blend and mixed at 2500 rpm for 5 min before melting it again in the VCM tool with the same configurations.

Physical mixture: 292.5 mg of PVA 4-88 was mixed with 7.5 mg of ARV-110 at 2500 rpm for 5 min in the mixer.

#### 2.2.2. Hot Melt Extrusion (HME)

For the first formulation, 58.5 g PVA 4-88 and 1.5 g ARV 110 were mixed in a Turbula T2F (WAB, Willy A. Bachofen AG, Muttenz, Switzerland) at 34 rpm for 20 min. The pre-mixed powder, including ARV 110 and PVA 4-88, was continuously fed into the Thermo Process HYG extruder feeder by using the twin-screw feeder. PVA 4-88 and ARV-110 were melted and mixed under barrel heating and twin-screwed shearing in the extruder (Pharma 11, Thermo Fisher, Waltham, MA, USA). The melt extrudate was cast on the cooling roll of a downstream sheet and taken off by using a 30 mm wide sheet die connected to the extruder.

The process parameters were adjusted, as illustrated in Table 1, to ensure that the film thickness was kept at about 80 µm.

For the second formulation, a plasticizer was added. 52.65 g PVA 4-88 and 1.5 g ARV-110 were mixed in a Turbula T2F (34 rpm, 20 min). The mixture was added to the hot melt extruder feeder. 5.85 g of triacetin was added to a beaker. The beaker with triacetin was connected to the HME tool through a peristaltic pump.

The pre-mixed powder, including ARV-110 and PVA 4-88, was continuously fed into the Thermo Process HYG extruder feeder by using the twin-screw feeder, and triacetin was continuously fed into the extruder by peristaltic pump model YZ1515X-B (Baoding Longer Precision Pump Co., Ltd., Baoding, China). The feeding speed of powder and triacetin are illustrated in Table 2. PVA 4-88, ARV 110, and triacetin were melted and mixed under barrel heating and twin-screwed shearing in the extruder. The resulting melt extrudate was cast on the cooling roll as described previously.

The process parameters were adjusted, as shown in Table 2, to ensure that the feeding speed of solid material (mixture of PVA 4-88 and ARV-110) and liquid material (Triacetin) were kept at about 9:1 proportion and the film thickness at around 80 µm.

#### 2.2.3. Solvent Casting

1.775 g of PVA (PVA 4-88 and PVA 5-88), 0.225 g of sorbitol, and 0.25 g of TWEEN^®^ 80 were mixed and dissolved in 10 mL of water. A minimal amount of Patent Blau 85 E131 was added to the placebo formulation for visual inspection of the films, and the mixture was stirred overnight at 100 rpm. This corresponds to the composition of the placebo formulations. One placebo formulation was achieved using 4-88 as PVA grade, and another one using 5-88. For the formulations containing the API, only the formulation containing PVA 4-88 was used. In this case, colorant was also not used due to the appearance of the ARV-110, which is yellow. Therefore, 13.5 mg of API was dissolved in 2.5 mL DMSO and added to the excipient mixture solution in a 1:2 (2.5 mL of API solution + 5 mL of polymer solution) ratio. In a second trial, the API concentration was increased using 28.8 mg of ARV-110, have been added to 2.5 mL of DMSO and subsequently mixed with 5 mL of polymer solution. The mixture was placed into a speed mixer for 3 min at 3200 rpm. The final solution was pipetted inside a casting geometry (Erichsen, Erichsen GmbH & Co. KG, Hemer, Germany) (see Figure S1 in the Supplementary Section) with a 400 µm thickness. After film casting, the final product was placed in a vacuum oven at 30 °C (<100 mbar). After a 16 h drying period, films were cut into 2 × 2 cm pieces for further analysis. The two placebo formulations, which compositions are highlighted in Table 3, are named 1Sc 2.5% (with PVA 4-88) and 2Sc 2.5% (with PVA 5-88). This is a name established and kept during the formulation development of the placebo ODFs, while 2.5% refers to the concentration of surfactant.

### 2.3. Analytical Characterization

#### 2.3.1. Differential Scanning Calorimetry (DSC)

DSC was performed using a DSC821e (Mettler Toledo, Gießen, Germany) and an N_2_ atmosphere (50.0 mL). The temperature scale was set from 30 °C to 320 °C with a heating rate of 10 °C/min.

This assessment was performed only with the ARV-110 as raw material to test its thermolability and, therefore, its suitability for the VCM method and, consequently, for the HME.

#### 2.3.2. X-Ray Powder Diffraction (XRPD)

Diffractograms of powders were measured using a Miniflex 600 X-ray diffractometer (Rigaku, Tokyo, Japan) with CuKα radiation (λ = 1.54 A). Samples were scanned in reflectance mode from 3° to 50° 2θ (deg), with a scan speed of 0.8° 2θ (deg)/min and a step size of 0.020° 2θ (deg). The acceleration voltage and current were 45 kV and 15 mA. This analysis was performed using vacuum-compressed and solvent-casted samples.

#### 2.3.3. Non-Sink Mini-Dissolution Test

For the samples gathered through VCM, the dissolution of the samples was determined at room temperature in phosphate buffer pH 6.8 as the dissolution medium. 5 mg of bulk drug or equivalent to 5 mg drug in milled VCM discs were weighed and placed into a 100 mL Erlenmeyer flask; subsequently, a magnetic stirring bar was added to the Erlenmeyer flask and stirred at 200 rpm, then 25 mL of dissolution medium was added (concentration: 0.2 mg/mL).

At predetermined time points (5, 10, 20, 40, 60, 90, 120 min), 500 µL of sample were withdrawn from the dissolution vessels and immediately replaced by 500 µL of fresh dissolution medium. The dissolution samples were then filtered through a 0.45 μm PTFE filter. The sample is diluted with Eluent B (acetonitrile + 0.1% formic acid) (1:1). Finally, the samples were analyzed towards drug content using high-performance liquid chromatography (HPLC).

The same test was performed with the extruded samples used as a sample for the test of 200 mg of ODF containing 5 mg of pure API. The same method has been used to perform the mini-dissolution test for the physical mixture containing PVA 4-88 and ARV-110. All measurements were performed in triplicates.

For the solvent-casted samples, a different non-sink mini-dissolution test has been performed using artificial saliva (a buffer solution (pH 6.8) containing 0.126 g/L NaCl, 0.964 g/L KCl, 0.189 g/L KSCN, 0.655 g/L KH_2_PO_4_, and 0.200 g/L urea) and elaborating a pH-shift.

##### Mini-Dissolution ODF and Raw Material

A 2 × 2 cm piece of film was dissolved in 0.5 mL artificial saliva. For the raw material, 5 mg of neat ARV-110 was placed in 0.5 mL of artificial saliva.

Next, 7.5 mL of 0.1 M HCl was added to the suspension and stirred at 300 rpm in the shaker TH15 (IKA, Breisgau, Germany) at 37 °C. After each sample was drawn, the dissolution medium was refilled using fresh 0.1 M HCl.

pH-shift: After 30 min, 2.0 mL of 0.2 M Na_3_PO_4_ × 12 H_2_O was added to the solution. After the samples were drawn, the dissolution was refilled using fresh 0.2 M Na_3_PO_4_ × 12 H_2_O.

The samples were filtered through a PTFE-filter (0.45 µm) and diluted with Eluent B (1:1). The sample volume was 500 µL, and the sample pickup time points were after 5, 10, 20, 35, 50, 60, 90, and 120 min. All samples were performed in triplicates.

#### 2.3.4. Reversed-Phase High-Performance Liquid Chromatography (RP-HPLC)

##### Content Assay

A 2 × 2 cm piece of film was weighed in a 10 mL volumetric flask and dissolved in 5 mL of DMSO. The solution was vortexed, and the sample volume increased to 10 mL using DMSO. After filtering the solution through a 0.45 µm PTFE filter, the sample was diluted 1:1 with Eluent B (acetonitrile + 0.1% formic acid) and placed in a vial for the RP-HPLC content determination.

An Agilent 1260 infinity HPLC system (Agilent, Santa Clara, CA, USA) equipped with an Agilent 1260 II variable wavelength detector was used. A C8-Column (Waters XBridge Column C8, 4.6 × 50 mm, 3.5 μm) column was used for the quantification. The injection volume was 5 μL, and the flow rate was 1.7 mL/min at 37 °C at the ratio illustrated in Table 4.

The wavelength was 254 nm. The eluents are composed as follows: Eluent A: 0.1% formic acid in water; Eluent B: 0.1% formic acid in acetonitrile. In this method, the calibration curve with concentrations from 100 µg/mL to 5 µg/mL was performed, reaching an R^2^ of 0.9999. The above-described HPLC method has been used for the dissolution test evaluation of all fabrication methods.

#### 2.3.5. Disintegration Test of ODF in Petri Dish

The film was cut into a 1.5 × 2 cm (for the extruded samples) piece or 2 × 2 cm (for the solvent cast samples) and placed into a petri dish containing 1 mL of artificial saliva. 4 mL of artificial saliva was added, and the petri dish was manually shaken to mimic the movement of the tongue. The time point at which the film was fully disintegrated was measured visually. Measurements were performed in triplicates. The test was applied for samples created with both HME and solvent casting.

#### 2.3.6. Tensile Strength Test

To measure the tensile strength, the texture analyzer TA.XTplusC Stable Micro Systems (Stable Micro Systems Ltd., Godalming, Surrey, UK) was used. The films were cut into 2 × 2 cm pieces, weighed, and the thickness measured. For thickness measurements, five different points of each film were measured, four on the corners and one in the center, with the Mitutoyo Digimatic Micrometer (Mitutoyo Corporation, Takatsu-ku, Kawasaki, Japan). The 2 × 2 cm piece of film was then positioned between the two probes of the instrument, which were consequently tightened to keep the film piece enclosed. The instrument started. During the test, the probes stretch the film, pulling both opposite lying edges in opposing directions until the film breaks. The probes used to perform this test, called the tensile strength test, are the miniature tensile grips A/MTG. The setting of the measurement set-up was a target distance of 2 cm with a test speed of 1 mm/s. The test is performed in triplicates for films with ARV-110 10× for the placebo formulations. The test was applied for samples created with both HME and solvent casting.

## 3. Results and Discussion

### 3.1. DSC Measurement

Preliminarily to the formulation development of the films, the thermostability of the API was tested through DSC measurements (see Figure S2 in the Supplementary Section) to evaluate which technologies would be applicable to the raw material. Once it was confirmed that the API tolerates formulation technologies that require high processing temperatures, it was subjected to VCM and HME in addition to solvent casting.

### 3.2. VCM

As ARV-110 displayed thermostability in a range used for our HME set-up, we chose to subject it to VCM. As the material availability of these PROTACs is limited, VCM offers the opportunity to perform small-scale screenings, which help to predict the substance`s performance in potential HME set-ups [12].

**Figure 1:**
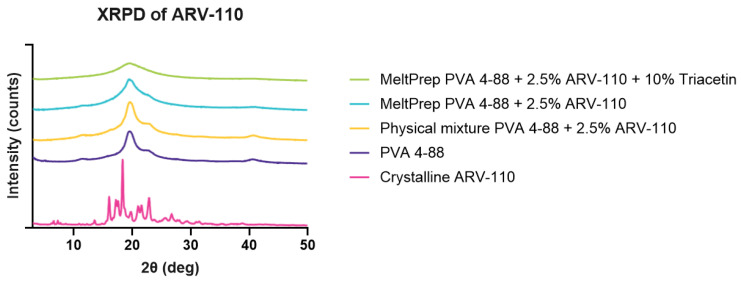
XRPD measurements of ARV-110 are crystalline, PVA 4-88, a physical mixture, and milled VCM discs. The *y*-axis represents an offset.

Plasticizers are important ingredients for formulations such as films and extrudates. In fact, they substantially aid in increasing their elastic properties. Interestingly, when used in formulations with PVA, plasticizers were proven to reduce the viscosity of different grades of the polymer. Additionally, they were able to expand the temperature-related processing window of this polymer, a pivotal support for formulating temperature-sensitive APIs [25]. The XRPD analysis of the VCM discs with and without the plasticizer triacetin was performed to assess the solid state of the samples (Figure 1).

The crystallinity of ARV-110 can be detected by sharp peaks at 16.08°, 18.35° and 22.98° 2θ in the diffractogram. These peaks were not observed for both VCM samples. However, this may be attributed to the very low percentage of API used (2.5%), which is likely below the limit of detection, which is supported by the diffractogram of the physical mixture, where no peaks related to the ARV-110 are visible. The broad peak at 20° observed in the physical mixture is related to PVA, which is a semi-crystalline polymer. Interestingly, these broad peaks are less pronounced in the VCM samples, indicating a decrease in crystallinity through processing. Comparing both VCM samples, the sample containing the plasticizer triacetin showed a more flattened peak, suggesting that the addition of plasticizer contributed to the reduction in the crystallinity of PVA.

**Figure 2:**
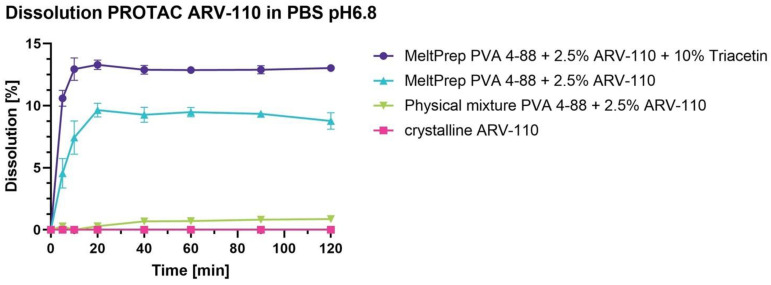
Non-sink dissolution of ARV-110, physical mixture with 2.5% ARV-110 + PVA 4-88, MeltPrep disk (milled) with 2.5% ARV-110 and PVA 4-88 and MeltPrep disk (milled) with 2.5% ARV-110, PVA 4-88 and triacetin 10% in PBS pH 6.8 at 100 rpm and room temperature.

The dissolution results showed an improvement in the drug dissolution rate when the ARV-110 is formulated in the VCM disc compared to the crude product (Figure 2).

Pure ARV-110 was not soluble, as no dissolved drug was detected over 120 min for the weakly basic PROTAC ARV-110 in PBS at pH 6.8. The sole presence of PVA 4-88 in the physical mixture enhanced dissolution slightly. This behavior was already introduced by Mareczek et al. when comparing spray-dried PVA-ARV-110 with the physical mixture of PVA and ARV-110 [24]. After VCM processing with PVA 4-88, a notable increase in dissolved ARV-110 was detected in the dissolution media. After 120 min, 15 µg/mL free drugs were detected. The addition of triacetin further increased dissolution, achieving 26 µg/mL after 120 min. The addition of triacetin increases the flexibility of films. More specifically, a study had shown that the presence of this plasticizer induces a reduction of the Tg and Tm, resulting in an increase of the segmental mobility of the PVA molecules, thus, better miscibility of the plasticizer in the polymeric film [26]. Yuksel et al. investigated Indomethacin loaded microspheres with and without triacetin, arriving at the conclusion that the addition of triacetin induced a better diffusion potential for the API molecules, thus resulting in an enhanced release of Indomethacin [27]. For both formulations, a supersaturated solution of ARV-110 was observed, reaching a c_max_ between 10 to 20 min. After this time point, the supersaturated state was maintained, illustrating the precipitation inhibition of the polymer PVA.

### 3.3. HME

With the positive results from our small-scale preliminary experiments, we decided to perform HME with ARV-110 and PVA. Both formulations, the binary mixture of API and polymer, as well as the ternary formulation containing triacetin, were used in this set-up.

The films without triacetin achieved a disintegration time of an average of ~121 ± 3 s (STDV n = 3) (see Table S1 in the Supplementary Section). Meanwhile, the disintegration tests of the ODF with triacetin performed better than without plasticizer, with an average of ~86 ± 4 s (STDV n = 3) (see Table S2 in the Supplementary Section). The faster the films disintegrate, the better they can be absorbed and follow the ADME path; simultaneously, the patient clearance increases. Again, this can be explained through the increased mobility by the addition of triacetin, thus increasing miscibility between all components and generating homogeneous films [26].

Following the protocol by Niese et al., an estimation of the yield point, the force at the yield offset, was calculated through the extrapolation of the stress–strain diagram [28].

The yield point is properly defined as the stress point at which a material begins to deform plastically. Prior to the yield point, the material will deform elastically, and when the applied stress is removed, it will return to its original shape. Once the yield point is passed, some fractions of the material will deform plastically, which means permanent and nonreversible deformation [29]. Therefore, the force required to reach the yield point provides valuable insights into the behavior of the films under stress. Testing the mechanical strength is crucial for three different aspects: the production or development of the product (it is important to ensure damage-free production), the packaging, and the handling of final consumers of the product [30].

The films fabricated through the HME method (see Figures S3 and S4 in the Supplementary Section) showed a high strength resistance in the texture analyzer measurements with a yield offset of 25.969 N ± 2.705 N (STDV n = 3), which indicates the development of a strong film [30]. The yield offset values obtained from our HME samples are comparable to those of paper (23.6 N) as described by Preis et al., and they simultaneously yield a discrete disintegration time of 86 s (Table S2, Supplementary Section). These films show a high resistance under stress, leading to little brittleness issues during development, production, and packaging and easy handling from patients.

**Figure 3:**
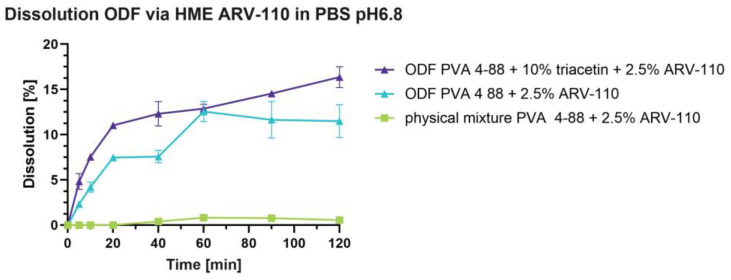
Non-sink dissolution of physical mixture of PVA 4-88 + 2.5% ARV-110, ODF with PVA 4-88 + 2.5% ARV-110, and ODF with PVA 4-88 + ARV-110 + 10% triacetin in PBS pH 6.8 at 100 rpm and room temperature.

Through preparing an ODF using HME, the dissolution of ARV-110 was increased up to ~16% of release for the ODF preparation containing 2.5% ARV-110 and 10% triacetin (Figure 3). Each piece of film was measured to contain around 1.8% ± 0.7% (STDV n = 5) for the formulation without triacetin and 2.3% ± 1.3% (STDV n = 5) for the formulation with triacetin of ARV-110 before dissolution. This formulation shows a good initial release of ARV-110 within the first 20 min, followed by a gradual increase over the dissolution measurement time. Also, the ODF preparation, containing only PVA 4-88 and 2.5% ARV-110, showed a substantial increase in dissolved drug compared to the physical mixture. After 120 min, the ODF without plasticizer reached around 13% and showed an overall slower release profile than the ODF with triacetin.

The films were prepared under two different temperature conditions, at 220 °C (without triacetin) and 190 °C (with 10% triacetin). Adding a plasticizer such as triacetin to the mixture allowed for a decrease in the operating temperature, which was also demonstrated in the literature to be beneficial for the stability of the API [31]. In the case of ARV-110, the processability using HME ran smoothly at both temperatures, and the ductility of ODF with triacetin was better than that without this plasticizer.

### 3.4. Solvent Casting

The solvent casting method used in this study followed a cold process formulation; therefore, no heat was introduced with the aim of designing a formulation method that can be used and transferred to thermolabile APIs.

The mixture consisted of a water-based 10 mL solution containing 17.75% of PVA 4-88 or PVA 5-88 as the polymer, 2.25% of sorbitol as plasticizer, and 2.5% of TWEEN^®^ 80 as surfactant with the addition of a minimal quantity of powder pigment Patent Blau 85 E131. The use of surfactant is crucial to facilitate the disintegration within the mouth and the fabrication of the films, giving a high output for the peeling of the film from the glass plate. The addition of Patent Blau facilitates handling and the determination of the disintegration time.

The results achieved with the disintegration test experiments showed an average of ~9 ± 2 s (STDV n = 3) (see Table S3, Supplementary Section) for the placebo formulation 1Sc 2.5% and ~14 ± 3 s (Table S4, Supplementary Section) (STDV n = 3) for the 2Sc 2.5% placebo formulation. These data suggested excellent disintegration properties of the films created with the solvent casting method, comparable to literature studies [32]. As we can see from Tables S3 and S4, the two formulations showed an increase in disintegration time for higher weights and thickness ranges. This characteristic will be again present in the following experiments with the API. Considering this behavior, it can be said that the two formulations achieved quite good results, and the longer disintegration time of the formulation with PVA 5-88 is easily attributable to the higher weights of the film pieces. However, the formulation containing PVA 4-88 as polymer resulted in lower standard deviations between the different samples, which, in the case of the weights, also confirms a better homogeneity of the film. For this reason, for the formulations containing the API, the 4-88 was used as the PVA grade of preference.

The disintegration times of the ODFs containing API reached an average value of ~18 ± 2 s (STDV n = 3) (Table S5, Supplementary Section) for the formulation 1Sc 2.5% containing 1.19% of API and ~35 ± 9 s (STDV n = 3) (Table S6, Supplementary Section) for the formulation containing 2.5% of API. The disintegration time is doubled compared to the placebo formulation (9 s for placebo versus 19 s for 1.9% API and 35 s for 2.5% API), which can also be attributed to the weights of the films that are also doubled (9 mg for placebo versus 19 mg for 1.19% and 2.5% API). The thickness range is also higher for the formulation with higher concentration.

Following the classification introduced by Gupta et al., the placebo, as well as the 1.19% ARV-110 ODFs, can be classified as Type 1 fast disintegrating ODFs with disintegration times < 30 s, while the formulation containing 2.5% API is at the threshold towards a moderate disintegration, which is defined as being >30 s [4].

**Figure 4:**
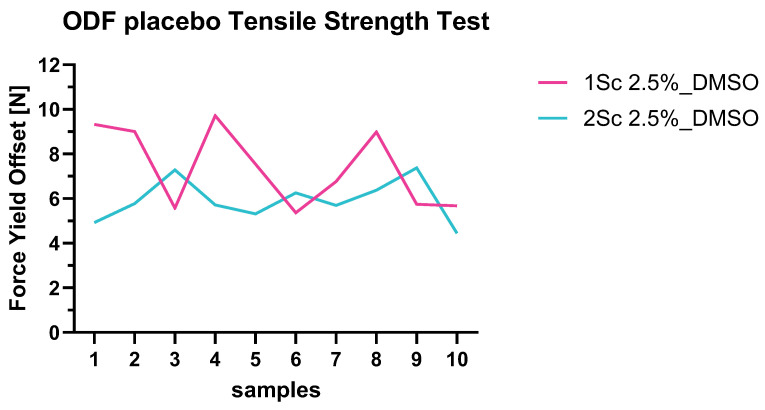
Force (N) at the yield offsets of the placebo formulations of ODFs through the solvent casting method.

Both placebo ODF formulations were investigated for their yield offset (Figure 4). The average forces of the yield offset are ~7.4 N ± 1.8 N for the PVA 4-88 formulation and ~ 5.9 N ± 0.9 N for the PVA 5-88 formulation.

For the formulations containing API, an average force at the yield offset of 6.5 N ± 0.5 N (STDV n = 3) for 1.19% API and an average of 8.7 N ± 0.3 N (STDV n = 3) for the formulation with the 2.5% of API were determined (Figure S5, Supplementary Section). The increase of the API concentration (which systematically corresponded to a higher weight of each film) influenced the strength of the films as well as the disintegration time. Therefore, films with higher weights and thickness ranges showed higher disintegration times and higher tensile strength. Looking at the yield strength values and comparing those to the forces over the distance of marketed products, these values are comparable and represent good strength characteristics. Preis et al. determined yield offset values for several marketed products to be between 1.48 and 7.18 N [30]. The herein-designed ARV-110 ODFs are, therefore, comparable to these values.

**Figure 5:**
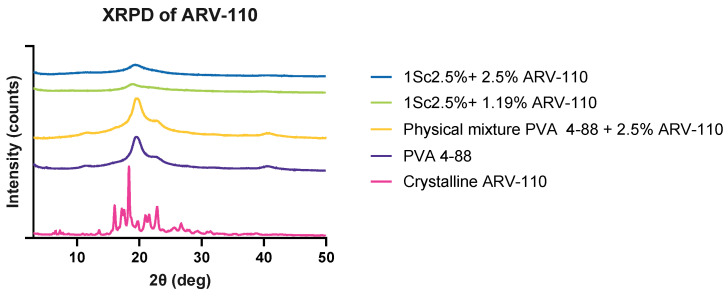
XRPD measurements of ARV-110 as crystalline and as ODFs. The *y*-axis represents an offset.

Both formulations, containing 1.19% and 2.5% ARV-110, show the halo in the XRPD with no indication of crystalline peaks, as seen for the raw ARV-110 (Figure 5). As discussed earlier, this is due to the low amount of API. The films also showed a broad peak at 20° related to PVA, which was less pronounced compared to raw PVA, also indicating a reduction of crystallinity in these samples.

In scientific literature, numerous in-vitro dissolution test assays are described for ODF preparations [33]. However, these assays do not faithfully replicate the genuine physiological conditions of the human body. A new dissolution test was developed to characterize the films using the solvent-casting method. Here, we assumed the disintegration of the ODF in saliva, followed by swallowing the dissolved formulation reaching the gastric and then the intestine. Therefore, we used artificial saliva for disintegration, followed by dissolution studies performed at pH 1.2 for 30 min, mimicking the gastric environment (HCl), with a pH shift to pH 6.8, mimicking the intestine (PBS).

**Figure 6:**
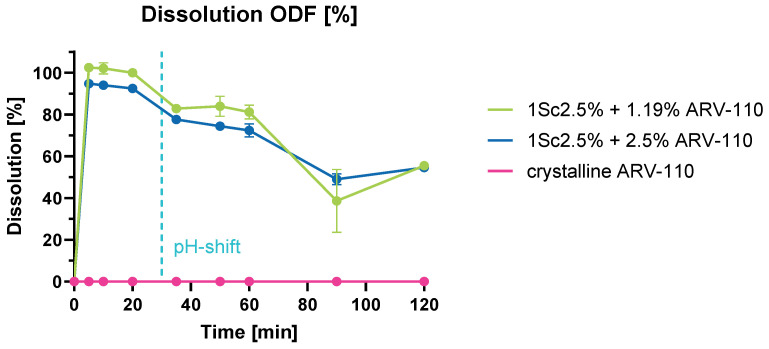
Graph of the dissolution tests of ARV-110 as crystalline, ODFs with 1.19% of ARV-110 and ODFs with 2.5% of ARV-110, with pH shift in PBS pH 6.8 at 100 rpm and 37 °C.

Figure 6 shows results from these dissolution experiments, indicating, again, the dissolution enhancement of ARV-110 through processing. The raw material, ARV-110, displays no measurable dissolution during the pH shift measurements, while for both formulations, a highly notable enhancement is determined. For the formulation containing a theoretical 1.19% API, the calculated amount of ARV-110 in the pieces of films previous to the dissolution test was 0.82% ± 0.09% (STDV n = 3). Meanwhile, for the formulation containing a theoretical 2.5% API, in the single pieces of films was calculated a 2.0% ± 0.067% (STDV n = 3) of ARV-110 before the dissolution test. Both formulations follow a similar dissolution profile over the pH-shift measurements and 120 min; in fact, even if they have a different concentration, the drug release profile is very similar, probably because between 1% and 2% of ARV-110 concentration, a saturation point in the physiological buffer is reached. With ARV-110 being a weakly basic API, dissolution is expected to be higher in acidic pH, which can be observed in Figure 6. With the change to pH 6.8, dissolution values decrease. However, in the case of ODFs through solvent casting in the buffer with pH 6.8, the drug release is much higher compared to the films through HME. Both ODF formulations can inhibit precipitation in PBS at pH 6.8.

Table 5 summarizes both 2.5% ARV-110 loaded ODFs prepared by either film casting or HME. The dissolution results obtained from HME and solvent casting cannot be directly compared due to variations in the test set-ups employed. Furthermore, XRPD characterization was not conducted for the HME samples, as the ODFs produced via solvent casting and HME were developed in different geographical locations. Lastly, the limited availability of the API utilized in this study necessitates further experimental validation of the results. We wish to emphasize these considerations as limitations of the study.

## 4. Conclusions

In conclusion, the study showed two different ways to produce ODFs using the PROTAC ARV-110 as API, overcoming the substantial obstacles of its poor solubility. The ODFs fabricated through HME yielded exceptionally robust films with disintegration times of 2 min (without plasticizer) and 90 s (including triacetin). Dissolution enhancement was identified through release studies in PBS at pH 6.8. Here, the formulation containing the plasticizer triacetin outperformed the formulation containing only PVA and API. HME, as a method that applies heat, was evaluated through a VCM screening process, which laid the basis for further formulation and process design. The solvent casting method created fast disintegrating films (disintegration around 30 s for both formulations, with 1.19% and 2.5% API), which reported lower strength values. In this case, the dissolution was dramatically enhanced, and a high supersaturation improvement was confirmed for the ODFs manufactured by film casting. In both cases, the dissolution performance of the drug increased compared to the crystalline form, creating a fundament for promising success and further exploration of ODFs for the delivery of PROTACs. Further research can be applied to advance the film casting process for the development of thicker films and greater payloads or to develop films with mucoadhesive properties to allow the PROTAC to be absorbed through the oral mucosa.

## Figures and Tables

**Table 1:** Process parameters of HME method for ODFs without triacetin fabrication.

Formulation		PVA 4-88 + 2.5% ARV-110
Process parameters Process parameters	Extruding Temperature (°C)	220
	Screw speed (rpm)	500
	Feeding speed (g/min)	4.94
	Haul-off speed (~m/min)	1.74
	Cooling roll temp (°C)	15
	Extruding Temperature (°C)	220

**Table 2:** Process parameters of HME method for ODFs with triacetin fabrication.

Formulation		PVA 4-88 + 10% Triacetin +2.5% ARV-110
Process parameters	Extruding Temperature (°C)	190
	Screw speed (rpm)	500
	Feeding speed (Solid/g/min)	5.01
	Feeding speed (Triacetin/g/min)	0.54
	Haul-off speed (~m/min)	1.74
	Cooling roll temp (°C)	15

**Table 3:** Placebo formulation composition of ODFs through solvent casting.

Ingredient	Content	Function
PVA 4-88 or PVA 5-88	Polyvinyl alcohol	Polymer
Sorbitol SI 150	Sorbitol	Plasticizer
TWEEN^®^ 80	Polysorbate 80	Surfactant
Water	H_2_O	Solvent

**Table 4:** Ratio of eluents within the HPLC method for non-sink mini dissolution test.

Time [min]	%A	%B	Flow [mL/min]
0.00	90	10	1.70
0.30	90	10	1.70
3.00	10	90	1.70
3.75	10	90	1.70
3.76	90	10	2.50
6.00	90	10	2.50

**Table 5:** Summary of characterization results of ODFs produced via HME and solvent casting containing a theoretical 2.5% ARV-110.

Analytics	HME (Form. with Triacetin)	Solvent Casting
Drug-load ARV-110 (%)	2.50	2.50
Thickness range (mm)	0.080–0.090	0.034–0.060
Disintegration time (s) ± STDV (n = 3)	86 ± 4	35 ± 9
Force at the Yield offset (N) ± STDV (n = 3)	25.969 ± 2.705	8.679 ± 0.342
Measured drug content (%) ± STDV (n = *)	2.27 ± 1.29 (* = 5)	2.0 ± 0.03 (* = 3)
Dissolution max (%) after 20 min ± STDV (n = 3)	11.01 ± 0.08	92.13 ± 0.33
Dissolution max (%) after 120 min ± STDV (n = 3)	16.36 ± 1.16	54.56 ± 1.69

## Data Availability

Data are contained within the article.

## Supplementary Materials

The following supporting information can be downloaded at: https://www.mdpi.com/article/10.3390/pharmaceutics16121499/s1, Figure S1: Erichsen film applicator geometry; Figure S2: DSC measurement of ARV-110, from 30 °C to 320 °C with a heating rate of 10 °C/min; Figure S3: Film roll with PROTAC ARV-110 2.5%; Figure S4: Film roll with PROTAC ARV-110 2.5% + Triacetin 10%; Figure S5: Force (N) at the yield offsets of the ODFs through solvent casting method with 2.5% and 1.19% of ARV-11; Table S1: Disintegration tests of films manufactured through HME with 2.5% ARV-110; Table S2: Disintegration tests of films manufactured through HME with 2.5% ARV-110 and 10% Triacetin; Table S3: Disintegration test results, weight and thickness (in triplicates) of the placebo formulation with PVA 4-88 of the solvent casted ODFs; Table S4: Disintegration test results, weight and thickness (in triplicates) of the placebo formulation with PVA 5-88 of the solvent casted ODFs; Table S5: Disintegration test results, weight, and thickness (in triplicates) of the ARV-110 1.19% formulation with PVA 4-88 of the solvent casted ODFs; Table S6: Disintegration test results, weight and thickness (in triplicates) of the ARV-110 2.5% formulation with PVA 4-88 of the solvent casted ODFs.

## References

[1] Musazzi U.M., Selmin F., Ortenzi M.A., Mohammed G.K., Franze S., Minghetti P., Cilurzo F. (2018). Personalized orodispersible films by hot melt ram extrusion 3D printing. Int. J. Pharm..

[2] Polonini H.C., Ferreira A.O., Raposo N.R.B., da Silva P., Brandao M.A.F. (2023). Compatibility Assessment of Novel Orodispersible Film Vehicle for Personalized Medicine with Selected Active Pharmaceutical Ingredients. J. Pers. Med..

[3] Scarpa M., Stegemann S., Hsiao W.K., Pichler H., Gaisford S., Bresciani M., Paudel A., Orlu M. (2017). Orodispersible films: Towards drug delivery in special populations. Int. J. Pharm..

[4] Gupta M.S., Kumar T.P., Gowda D.V. (2020). Orodispersible Thin Film: A new patient-centered innovation. J. Drug Deliv. Sci. Technol..

[5] Hoffmann E.M., Breitenbach A., Breitkreutz J. (2011). Advances in orodispersible films for drug delivery. Expert. Opin. Drug Deliv..

[6] Borges A.F., Silva C., Coelho J.F., Simoes S. (2015). Oral films: Current status and future perspectives: I-Galenical development and quality attributes. J. Control Release.

[7] Salawi A. (2022). An Insight into Preparatory Methods and Characterization of Orodispersible Film-A Review. Pharmaceuticals.

[8] Visser J.C., Woerdenbag H.J., Crediet S., Gerrits E., Lesschen M.A., Hinrichs W.L.J., Breitkreutz J., Frijlink H.W. (2015). Orodispersible films in individualized pharmacotherapy: The development of a formulation for pharmacy preparations. Int. J. Pharm..

[9] Parikh T., Gupta S.S., Meena A.K., Vitez I., Mahajan N., Serajuddin A.T. (2015). Application of film-casting technique to investigate drug-polymer miscibility in solid dispersion and hot-melt extrudate. J. Pharm. Sci..

[10] Patil H., Tiwari R.V., Repka M.A. (2016). Hot-Melt Extrusion: From Theory to Application in Pharmaceutical Formulation. AAPS PharmSciTech.

[11] Tumuluri V.S., Kemper M.S., Lewis I.R., Prodduturi S., Majumdar S., Avery B.A., Repka M.A. (2008). Off-line and on-line measurements of drug-loaded hot-melt extruded films using Raman spectroscopy. Int. J. Pharm..

[12] Shadambikar G., Kipping T., Di-Gallo N., Elia A.G., Knuttel A.N., Treffer D., Repka M.A. (2020). Vacuum Compression Molding as a Screening Tool to Investigate Carrier Suitability for Hot-Melt Extrusion Formulations. Pharmaceutics.

[13] Kayser K., Monschke M., Wagner K.G. (2022). ASD Formation Prior to Material Characterization as Key Parameter for Accurate Measurements and Subsequent Process Simulation for Hot-Melt Extrusion. AAPS PharmSciTech.

[14] Monschke M., Kayser K., Wagner K.G. (2020). Processing of Polyvinyl Acetate Phthalate in Hot-Melt Extrusion-Preparation of Amorphous Solid Dispersions. Pharmaceutics.

[15] Li H., Zhang M., Xiong L., Feng W., Williams R.O. (2020). Bioavailability Improvement of Carbamazepine via Oral Administration of Modified-Release Amorphous Solid Dispersions in Rats. Pharmaceutics.

[16] Skolakova T., Slamova M., Skolakova A., Kaderabkova A., Patera J., Zamostny P. (2019). Investigation of Dissolution Mechanism and Release Kinetics of Poorly Water-Soluble Tadalafil from Amorphous Solid Dispersions Prepared by Various Methods. Pharmaceutics.

[17] Xie T., Taylor L.S. (2016). Dissolution Performance of High Drug Loading Celecoxib Amorphous Solid Dispersions Formulated with Polymer Combinations. Pharm. Res..

[18] Zecevic D.E., Meier R., Daniels R., Wagner K.G. (2014). Site specific solubility improvement using solid dispersions of HPMC-AS/HPC SSL—Mixtures. Eur. J. Pharm. Biopharm..

[19] Zhang Y., Zhang Y., Zhang Y. (2022). The Role of Natural Products in the Treatment of Alzheimer’s Disease: A Review. Molecules.

[20] Lai A.C., Crews C.M. (2017). Induced protein degradation: An emerging drug discovery paradigm. Nat. Rev. Drug Discov..

[21] Yang W., Gadgil P., Krishnamurthy V.R., Landis M., Mallick P., Patel D., Patel P.J., Reid D.L., Sanchez-Felix M. (2020). The Evolving Druggability and Developability Space: Chemically Modified New Modalities and Emerging Small Molecules. AAPS J..

[22] Postges F., Kayser K., Appelhaus J., Monschke M., Gutschow M., Steinebach C., Wagner K.G. (2023). Solubility Enhanced Formulation Approaches to Overcome Oral Delivery Obstacles of PROTACs. Pharmaceutics.

[23] Hofmann N., Harms M., Mader K. (2024). ASDs of PROTACs: Spray-dried solid dispersions as enabling formulations. Int. J. Pharm..

[24] Mareczek L., Mueller L.K., Halstenberg L., Geiger T.M., Walz M., Zheng M., Hausch F. (2024). Use of Poly(vinyl alcohol) in Spray-Dried Dispersions: Enhancing Solubility and Stability of Proteolysis Targeting Chimeras. Pharmaceutics.

[25] Hess F., Kipping T., Weitschies W., Krause J. (2024). Understanding the Interaction of Thermal, Rheological, and Mechanical Parameters Critical for the Processability of Polyvinyl Alcohol-Based Systems during Hot Melt Extrusion. Pharmaceutics.

[26] Zuber S.A., Rusli A., Ismail H. (2019). Effectiveness of triacetin and triethyl citrate as plasticizer in polyvinyl alcohol. Proc. Mater. Today: Proc..

[27] Yuksel N., Baykara M., Shirinzade H., Suzen S. (2011). Investigation of triacetin effect on indomethacin release from poly(methyl methacrylate) microspheres: Evaluation of interactions using FT-IR and NMR spectroscopies. Int. J. Pharm..

[28] Niese S., Quodbach J. (2019). Formulation development of a continuously manufactured orodispersible film containing warfarin sodium for individualized dosing. Eur. J. Pharm. Biopharm..

[29] Vaidya A., Pathak K. (2019). 17—Mechanical stability of dental matererials. Appl. Nanocompos. Mater. Dent..

[30] Preis M., Knop K., Breitkreutz J. (2014). Mechanical strength test for orodispersible and buccal films. Int. J. Pharm..

[31] Desai D., Sandhu H., Shah N., Malick W., Zia H., Phuapradit W., Vaka S.R.K. (2018). Selection of Solid-State Plasticizers as Processing Aids for Hot-Melt Extrusion. J. Pharm. Sci..

[32] Jacob S., Boddu S.H.S., Bhandare R., Ahmad S.S., Nair A.B. (2023). Formulation and Characterization of Orodispersible Films for the Delivery of Anticancer Drugs. Pharmaceutics.

[33] Krampe R., Sieber D., Pein-Hackelbusch M., Breitkreutz J. (2016). A new biorelevant dissolution method for orodispersible films. Eur. J. Pharm. Biopharm..

