# Supplementary material for: Exploring Orodispersible Films Containing the Proteolysis Targeting Chimera ARV-110 in Hot Melt Extrusion and Solvent Casting Using Polyvinyl Alcohol
Source: Pharmaceutics. 2024 Nov 22;16(12):1499. doi: 10.3390/pharmaceutics16121499 (PMC11678735; doi:10.3390/pharmaceutics16121499)
Supplement: Supplementary file 1 [file pharmaceutics-16-01499-s001.zip › pharmaceutics-3256862-supplementary.pdf]

pharmaceutics

Supplementary Materials:

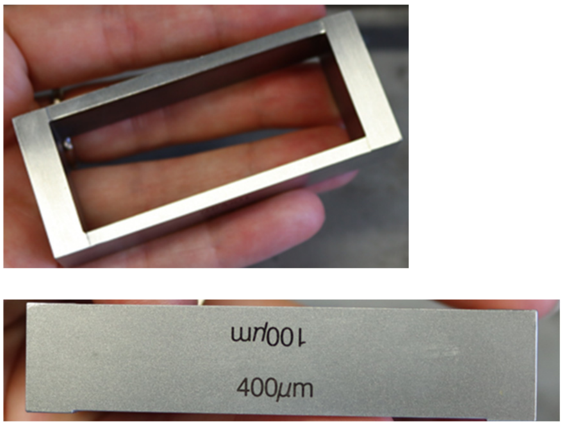

Figure S1: Erichsen film applicator geometry

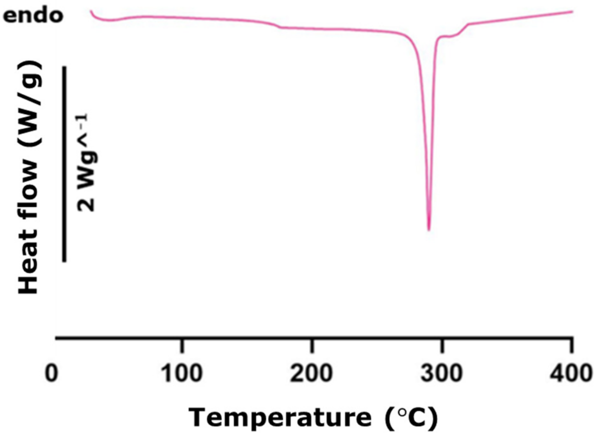

Figure S2: DSC measurement of ARV-110, from 30 °C to 320 °C with a heating rate of 10 °C/min..

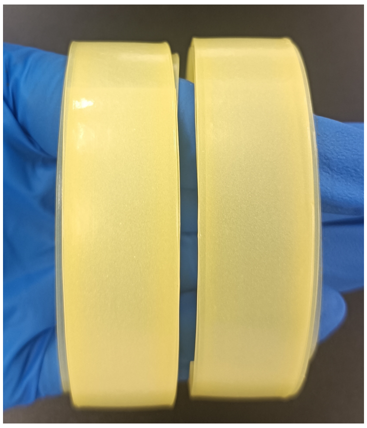

Figure S3: Film roll with PROTAC ARV-110 2.5%

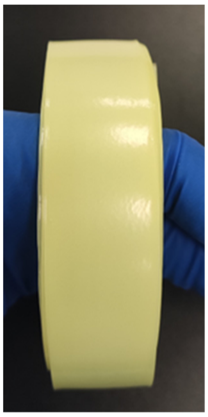

Figure S4. Film roll with PROTAC ARV-110 2.5% + Triacetin 10%

Table S1: Disintegration tests of films manufactured through HME with 2.5% ARV-110.

ODFs with ARV-110 2.5% through HME without Triacetin		
Sample	Disintegration time (sec)	Thickness range (mm)
1	123.00	0.080-0.090
2	124.00	0.080-0.090
3	118.00	0.080-0.090
mean value	121.67	
STDV	3.21	

Table S2: Disintegration tests of films manufactured through HME with 2.5% ARV-110 and 10% Triacetin.

ODFs with ARV-110 2.5% through HME with Triacetin		
Sample	Disintegration time (sec)	Thickness range (mm)
1	82.00	0.080-0.090
2	90.00	0.080-0.090
3	86.00	0.080-0.090
mean value	86.00	
STDV	4.00	

Table S3: Disintegration test results, weight and thickness (in triplicates) of the placebo formulation with PVA 4-88 of the solvent casted ODFs

1Sc 2.5 % placebo			
Sample	Weight (mg)	Disintegration time (sec)	Thickness range (mm)
1	7.64	8.00	0.023-0.030
2	8.24	8.00	0.022-0.031
3	10.06	12.00	0.023-0.029
mean value	8.65	9.33	
STDV	1.26	2.31	

Table S4: Disintegration test results, weight and thickness (in triplicates) of the placebo formulation with PVA 5-88 of the solvent casted ODFs

2Sc 2.5 % placebo			
Sample	Weight (mg)	Disintegration time (sec)	Thickness range (mm)
1	16.88	18.00	0.026-0.040
2	13.43	12.00	0.022-0.029
3	11.45	14.00	0.020-0.035
mean value	13.92	14.67	
STDV	2.75	3.06	

Table S5: Disintegration test results, weight, and thickness (in triplicates) of the ARV-110 1.19 % formulation with PVA 4-88 of the solvent casted ODFs

1Sc 2.5 % with ARV-110 1.19 %			
Sample	Weight (mg)	Disintegration time (sec)	Thickness range (mm)
1	19.00	18.00	0.035-0.041
2	20.07	20.00	0.038-0.042
3	17.30	16.00	0.039-0.043
mean value	18.79	18.00	
STDV	1.40	2.00	

Table S6: Disintegration test results, weight and thickness (in triplicates) of the ARV-110 2.5 % formulation with PVA 4-88 of the solvent casted ODFs

1Sc 2.5% with ARV-110 2.5%			
Sample	Weight (mg)	Disintegration time (sec)	Thickness range (mm)
1	16.56	45.00	0.036-0.045
2	21.48	32.00	0.034-0.049
3	18.03	28.00	0.041-0.060
mean value	18.69	35.00	
STDV	2.53	8.89	

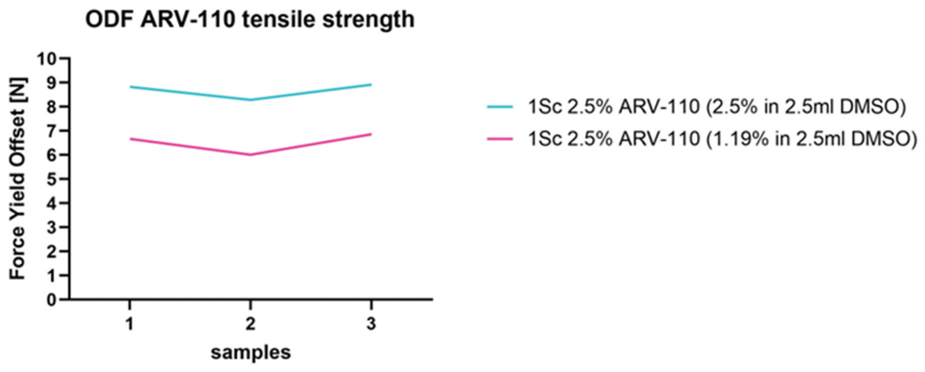

Figure S5: Force (N) at the yield offsets of the ODFs through solvent casting method with 2.5 % and 1.19 % of ARV-11.
